# RECURRENCE IN PN0 GASTRIC CANCER: RISK FACTORS IN THE OCCIDENT

**DOI:** 10.1590/0102-672020210001e1562

**Published:** 2021-05-14

**Authors:** Karolyne Ernesto Luiz NOBRE, Marina Alessandra PEREIRA, Marcus Fernando Kodama Pertille RAMOS, Ulysses RIBEIRO, Bruno ZILBERSTEIN, Ivan CECCONELLO, André Roncon DIAS

**Affiliations:** 1 School of Medical Sciences, UNIFACISA, Medicine, Campina Grande, PB, Brazil; 2 Cancer Institute, Hospital das Clinicas, University of São Paulo, São Paulo, SP, Brazil

**Keywords:** Stomach neoplasms, Gastrectomy, Prognosis, Survival analysis, Neoplasias gástricas, Gastrectomia, Prognóstico, Análise de sobrevida

## Abstract

**Background:**

Nearly 10% of node negative gastric cancer patients who underwent curative surgery have disease recurrence. Western data is extremely poor on this matter and identifying the risk factors that associate with relapse may allow new strategies to improve survival.

**Aim:**

Verify the clinical and pathological characteristics that correlate with recurrence in node negative gastric cancer.

**Methods:**

All gastric cancer patients submitted to gastrectomy between 2009 and 2019 at our institution and pathologically classified as N0 were considered. Their data were available in a prospective database. Inclusion criteria were: gastric adenocarcinoma, node negative, gastrectomy with curative intent, R0 resection. Main outcomes studied were: disease-free survival and overall survival.

**Results:**

A total of 270 patients fulfilled the inclusion criteria. Mean age was 63-year-old and 155 were males. Subtotal gastrectomy and D2 lymphadenectomy were performed in 64% and 74.4%, respectively. Mean lymph node yield was 37.6. Early GC was present in 54.1% of the cases. Mean follow-up was 40.8 months and 19 (7%) patients relapsed. Disease-free survival and overall survival were 90.9% and 74.6%, respectively. Independent risk factors for worse disease-free survival were: total gastrectomy, lesion size ≥3.4 cm, higher pT status and <16 lymph nodes resected.

**Conclusion:**

In western gastric cancer pN0 patients submitted to gastrectomy, lymph node count <16, pT3-4 status, tumor size ≥3.4 cm, total gastrectomy and presence of lymphatic invasion, are all risk factors for disease relapse.

## RESUMO

**Racional:**

Aproximadamente 10% dos pacientes com câncer gástrico submetidos a operação curativa e sem linfonodos acometidos irão apresentam recorrência da doença. Os dados ocidentais são extremamente pobres sobre este assunto e a identificação dos fatores de risco associados à recidiva podem permitir novas estratégias para melhorar a sobrevida.

**Objetivo:**

Identificar as características clínicas e patológicas que se correlacionam com recidiva em pacientes com câncer gástrico pN0.

**Métodos:**

Foram considerados todos os pacientes com câncer gástrico submetidos à gastrectomia entre 2009 e 2019 em nossa instituição e que na classificação patológica não apresentaram acometimento linfonodal. Os critérios de inclusão foram: adenocarcinoma gástrico, pN0, gastrectomia com intenção curativa, ressecção R0. Os principais desfechos estudados foram: sobrevida livre de doença e sobrevida global.

**Resultados:**

Ao todo 270 pacientes preencheram os critérios de inclusão. A idade média foi de 63 anos e 155 eram homens. A gastrectomia subtotal e a linfadenectomia D2 foram realizadas em 64% e 74,4%, respectivamente. A média de linfonodos ressecados foi de 37,6. Câncer gástrico precoce estava presente em 54,1% dos casos. O seguimento médio foi de 40,8 meses e 19 (7%) apresentaram recidiva. A sobrevida livre de doença e sobrevida global foram de 90,9% e 74,6%, respectivamente. Os fatores de risco independentes para pior sobrevida livre de doença foram: gastrectomia total, lesão ≥3,4 cm, status pT avançado e <16 linfonodos ressecados.

**Conclusão:**

Os fatores de risco para recidiva no grupo estudado foram: <16 linfonodos ressecados, status pT3-4, tumor ≥3,4 cm, gastrectomia total e presença de invasão linfática.

## INTRODUCTION

Gastric cancer (GC) has high prevalence worldwide and is a major cause of cancer-related death^2^ ^,^ ^10^ ^,^ ^20^. Lymph node metastasis is the most important prognostic factor^18^ ^,^ ^23^ ^,^ ^29^; however, nearly 10% of pN0 patients who underwent gastrectomy have disease recurrence^18^ ^,^ ^22^ ^,^ ^23^ ^,^ ^29^. This particular subgroup may benefit from adjuvant therapy and more intensive follow-up^22,26, 28^). Nevertheless, the risk factors associated with recurrence in this population are poorly reported, especially in the western world.

The aim of this study was to evaluate the clinical and pathological characteristics related to recurrence in pN0 GC patients who underwent curative gastrectomy.

## METHODS

The present study was approved by our ethics committee and is registered online at plataformabrasil.saude.gov.br under CAAE: 62915516.2.0000.0065.

### Patients

All GC patients submitted to gastrectomy with curative intent between 2009 and 2019 at the Instituto do Cancer de São Paulo, São Paulo, SP, Brazil were considered. Data were available in a prospective database. Inclusion criteria were: gastric adenocarcinoma, absence of lymph node metastasis (pN0), gastrectomy with D1 or D2 lymphadenectomy. Palliative surgery and patients with postoperative mortality were excluded from the analysis. 

Collected clinical characteristic included: age, gender, preoperative laboratory tests (hemoglobin, albumin, neutrophil/lymphocyte ratio), lymphadenectomy performed, pathological report, presence of comorbidities (Charlson classification)^5^.

The number of lymph nodes dissected was evaluated according to the minimum values recommended for examination by the American Joint Committee on Cancer and the Japanese Gastric Cancer Association (at least 16 and 25, respectively)^1^ ^,^ ^13^.

Postoperative complications were graded by Clavien-Dindo classification, and Clavien >II were considered as major ones^8^.

Total or subtotal gastrectomy and lymphadenectomy extension were performed according to the Japanese Guidelines^13^. Specimens were fixed in Carnoy’s solution and pathologic analysis followed the recommendations of the College of American Pathologists^6^ ^,^ ^24^. The 8^th^ edition of the TNM was used for staging^1^.

### Statistical analysis

Chi-square or Fisher exact tests were used for qualitative variables and t-student test for quantitative ones. Receiver Operating Characteristic (ROC) curve was used to determine the cut-off value for tumor size that correlated with disease recurrence. The area under the curve (AUC) was employed as a measure of accuracy. Overall survival and disease-free survival were estimated by the Kaplan-Meier test and differences examined by the log rank test. Survival was determined from the day of the surgery until death, recurrence or the or date of last contact. Variables independently affecting prognosis were investigated by multivariate analysis using the Cox proportional hazards model. Variables with p<0.100 univariate analysis were included in the multivariate model. SPSS was used for statistical analysis. All tests were two-sided and p<0.05 was considered significant.

## RESULTS

**FIGURE 1:**
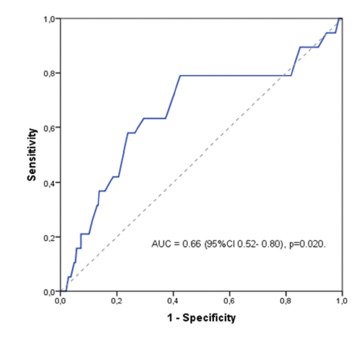
Receiver operating characteristic (ROC) curve for tumor size related with recurrence.

A total of 270 patients fulfilled the inclusion criteria. Mean age was 63.6-year-old (23-89) and 155 (57.4%) were males. Subtotal gastrectomy and D2 lymphadenectomy were performed in 64% and 74.4%, respectively. Mean lymph node yield was 37.6. Early GC (mucosa and submucosa) was present in 54.1% of cases. The mean tumor size was 3 cm (0.2-14), and the cutoff value obtained by the ROC curve was 3.4 cm, with an accuracy of 66% (Figure 1).

In a mean follow-up was 40.8 months, 19 (7%) patients had disease recurrence. The characteristics of non-recurrence and recurrence groups are summarized in Table 1.

**TABLE 1:** Clinical and pathological characteristics of gastric cancer pN0 patients with and without disease recurrence (n=270)

Variables		Non-recurrence n=251 (%)	Recurrence n=19 (%)	p
Gender				0.662
	Male	145 (57.8)	10 (52.6)	
	Female	106 (42.2)	9 (47.4)	
Age (years)				0.144
	Mean (SD)	63.9 (12.5)	59.6 (12.7)	
Hemoglobin (g/dl)				0.667
	Mean (SD)	13.2 (9.4)	12.1 (1.7)	
Albumin (g/dl)				0.560
	Mean (SD)	4.1 (0.6)	4.0 (0.5)	
Neutrophil lymphocyte ratio (NLR)				0.487
	Mean (SD)	2.54 (1.90)	2.20 (0.87)	
Charlson-Deyo Comorbidity Index (CCI)				0.820
	0	165 (65.7)	12 (63.2)	
	≥1	86 (34.3)	7 (36.8)	
ASA classification				1.0
	I / II	203 (80.9)	16 (84.2)	
	III / IV	48 (19.1)	3 (15.8)	
Type of resection				0.002
	Subtotal	167 (66.5)	6 (31.6)	
	Total	84 (33.5)	13 (68.4)	
Type of lymphadenectomy				0.276
	D1	62 (24.7)	7 (36.8)	
	D2	189 (75.3)	12 (63.2)	
Tumor size (cm)				0.002
	<3.4	141 (57.1)	4 (21.1)	
	≥ 3.4	106 (42.9)	15 (78.9)	
Lauren type				0.144
	Intestinal	161 (64.1)	9 (47.4)	
	Diffuse/mixed	90 (35.9)	10 (52.6)	
Histological grade				0.499
	Well/moderately differentiated	139 (55.4)	9 (47.4)	
	Poorly differentiated	112 (44.6)	10 (52.6)	
Lymphatic invasion				0.049
	No	197 (78.5)	11 (57.9)	
	Yes	54 (21.5)	8 (42.1)	
Venous invasion				0.726
	No	218 (86.9)	16 (84.2)	
	Yes	33 (13.1)	3 (15.8)	
Perineural invasion				0.139
	No	201 (80.1)	12 (63.2)	
	Yes	50 (19.9)	7 (36.8)	
pT status				0.004
	pT1	142 (56.6)	4 (21.1)	
	pT2	40 (15.9)	3 (15.8)	
	pT3	55 (21.9)	8 (42.1)	
	pT4	14 (5.6)	4 (21.1)	
Lymph node count				0.585
	Mean (SD)	37.8 (18.5)	35.4 (22.6)	
Number of lymph nodes				0.401
	<25	57 (22.7)	6 (31.6)	
	>25	194 (77.3)	13 (68.4)	
Number of lymph nodes				0.085
	<16	21 (8.4)	4 (21.1)	
	>16	230 (91.6)	15 (78.9)	
pTNM status				0.002
	I	181 (72.1)	6 (31.6)	
	II	64 (25.5)	11 (57.9)	
	III	6 (2.4)	2 (10.5)	

SD=standard deviation; ASA=American Society of Anesthesiologists; p*-*values in bold are statistically significant.

Total gastrectomy (p=0.002), larger tumor size (p=0.002), presence of lymphatic invasion (p=0.049) and advanced pT status (p=0.004) were associate with recurrence patients. There was no difference between the groups regarding the histological type, extension of lymphadenectomy and the number of lymph nodes dissected.

The length of hospital stay was similar between the groups (10.9 and 15.4 days for non-recurrence and recurrence group, respectively; p=0.106). Major complications occurred in 6.3% of the cases (5.6% and 3% for non-recurrence and recurrence group, respectively; p=0.107).

### Survival analysis

**FIGURE 2:**
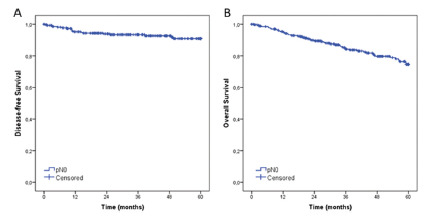
Disease-free survival and overall survival for pN0 gastric cancer patients (n=270).

Disease-free survival and overall survival rates for the entire population were 90.9% and 74.6%, respectively (Figure 2).

**FIGURE 3:**
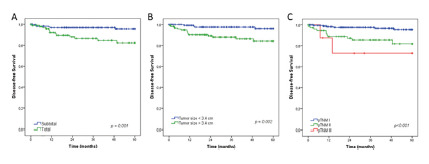
Disease-free survival according to extent of surgery, lesion size and pTNM

Considering the extent of resection, pN0 GC patients who underwent total gastrectomy had worse disease-free survival compared with subtotal gastrectomy (p=0.001). Also, based on the cutoff for tumor size, lesions ≥3.4 cm had a significantly poorer survival than smaller lesions (p=0.002). disease-free survival was worse according to the pTNM (p<0.001, Figure 3).

In multivariate analysis, total gastrectomy, tumor size ≥ 3.4 cm, advanced pT status and <16 lymph nodes resected were independent risk factors for worse disease-free survival (Table 2).

**TABLE 2:** Univariate and multivariate analysis for disease-free survival in pN0 gastric cancer patients

Disease-free suvival	Univariate			Multivariate		
Variables	HR	95%CI	p	HR	95%CI	p
Male (vs. female)	0.82	0.33 - 2.03	0.671	-	-	-
Age ≥ 65 (vs. <)	0.59	0.23 - 1.50	0.269	-	-	-
Total gastrectomy (vs. subtotal)	4.23	1.61 - 11.13	0.004	3.63	1.37 - 9.59	0.009
Diffuse/mixed type (vs. intestinal)	1.91	0.78 - 4.71	0.157	-	-	-
Tumor size ≥ 3.4 cm (vs. <)	4.95	1.64 - 14.94	0.005	3.50	1.51 - 16.58	0.040
pT3/T4 status (vs. pT1/T2)	4.31	1.69 - 10.95	0.002	3.65	1.26 - 10.55	0.017
<16 LNs (vs. ≥)	2.78	0.92 - 8.40	0.069	5.01	1.51 - 16.58	0.008

CI=confidence interval; HR=hazard ratio; LN=lymph node; variables with p<0.100 were included in the multivariate analysis; p-values in bold are statistically significant.

## DISCUSSION

Following gastrectomy with curative intent, pN0 GC patients have good prognosis, even if they were N+ before an eventual neoadjuvant therapy^2^ ^,^ ^21^. For those with advanced stage (T3-4 lesions) adjuvant therapy is indicated, but this is not usually recommended for the rest and some of them will relapse. By identifying this subgroup of patients, we may intensify their follow-up and/or refer them for adjuvant treatment, hoping to extend their survival. Besides the pT status ^3^ ^,^ ^7^ ^,^ ^15^, inadequate lymphadenectomy^3^ ^,^ ^15^, low number of lymph nodes examined^11, 12,14,26^, diffuse histology^16^ ^,^ ^25^ and neural or lymphatic invasion^6^ ^,^ ^13^ ^,^ ^14^ ^,^ ^16^ ^,^ ^30^ have been correlated with recurrence.

In our cohort, relapse occurred in 7% of the studied population and, besides pT and lymphatic invasion, total gastrectomy and lesions ≥3.4 cm also correlated with disease recurrence. Larger tumors require total gastrectomy more frequently, so the type of surgery must be considered in this context. Tumor size has been described as a predictor of prognosis in GC; however, there is no consensus on the cut-off value^17^ ^,^ ^27^ ^,^ ^31^. 

Lymphatic invasion is considered a high-risk feature^9^, so that adjuvant therapy may be recommended for pT2N0 patients^12^ ^,^ ^15^ ^,^ ^19^. In its presence we strongly advice additional investigation, with further cuts and immunohistochemical analysis of the lymph nodes retrieved^6^ ^,^ ^13^ ^,^ ^22^.

Low lymph node count (<16) was more frequent in the recurrence group (8.4% vs. 21.1%), however with no significance, probably due to the low number of patients with recurrence. At multivariate analysis it was the most important independent risk factor for worse disease-free survival (HR=5).

The main limitations of our study are its retrospective nature and the low number of patients with relapse. Nevertheless, to assess the real impact of the studied factors in survival, palliative procedures, R1/2 resections and stage IV patients were not included. Lymph node count was also high certifying that adequate staging as performed.

Also, available western data concerning recurrence in pN0 GC is extremely poor^4^; so, our findings provide further evidence to help guide decision in the studied population.

## CONCLUSION

In western GC pN0 patients submitted to gastrectomy with curative intent, lymph node count <16, pT3-4 status, tumor size ≥3.4 cm, total gastrectomy and presence of lymphatic invasion are all risk factors for disease relapse.
